# Socio-cultural influences on the behaviour of South Asian women with diabetes in pregnancy: qualitative study using a multi-level theoretical approach

**DOI:** 10.1186/s12916-015-0360-1

**Published:** 2015-05-21

**Authors:** Trisha Greenhalgh, Megan Clinch, Nur Afsar, Yasmin Choudhury, Rita Sudra, Desirée Campbell-Richards, Anne Claydon, Graham A. Hitman, Philippa Hanson, Sarah Finer

**Affiliations:** Nuffield Department of Primary Care Health Sciences, University of Oxford, New Radcliffe House, Walton St, Oxford, OX2 6GG UK; Blizard Institute, Barts and the London School of Medicine and Dentistry, London, E1 2AT UK; Community Health and Social Medicine Department, Sophie Davis School of Biomedical Education, City University of New York Medical School, New York, 10031 USA; Department of Diabetes, Newham University Hospital, Glen Road, Plaistow, London, E13 8SL UK; Department of Diabetes, Royal London Hospital, Whitechapel Rd, London, E1 1BB UK

**Keywords:** Diabetes, Fetal programming, Gestational diabetes, Illness narrative, Pregnancy, Social ecology

## Abstract

**Background:**

Diabetes in pregnancy is common in South Asians, especially those from low-income backgrounds, and leads to short-term morbidity and longer-term metabolic programming in mother and offspring. We sought to understand the multiple influences on behaviour (hence risks to metabolic health) of South Asian mothers and their unborn child, theorise how these influences interact and build over time, and inform the design of culturally congruent, multi-level interventions.

**Methods:**

Our sample for this qualitative study was 45 women of Bangladeshi, Indian, Sri Lankan, or Pakistani origin aged 21–45 years with a history of diabetes in pregnancy, recruited from diabetes and antenatal services in two deprived London boroughs. Overall, 17 women shared their experiences of diabetes, pregnancy, and health services in group discussions and 28 women gave individual narrative interviews, facilitated by multilingual researchers, audiotaped, translated, and transcribed. Data were analysed using the constant comparative method, drawing on sociological and narrative theories.

**Results:**

Key storylines (over-arching narratives) recurred across all ethnic groups studied. Short-term storylines depicted the experience of diabetic pregnancy as stressful, difficult to control, and associated with negative symptoms, especially tiredness. Taking exercise and restricting diet often worsened these symptoms and conflicted with advice from relatives and peers. Many women believed that exercise in pregnancy would damage the fetus and drain the mother’s strength, and that eating would be strength-giving for mother and fetus. These short-term storylines were nested within medium-term storylines about family life, especially the cultural, practical, and material constraints of the traditional South Asian wife and mother role and past experiences of illness and healthcare, and within longer-term storylines about genetic, cultural, and material heritage – including migration, acculturation, and family memories of food insecurity. While peer advice was familiar, meaningful, and morally resonant, health education advice from clinicians was usually unfamiliar and devoid of cultural meaning.

**Conclusions:**

‘Behaviour change’ interventions aimed at preventing and managing diabetes in South Asian women before and during pregnancy are likely to be ineffective if delivered in a socio-cultural vacuum. Individual education should be supplemented with community-level interventions to address the socio-material constraints and cultural frames within which behavioural ‘choices’ are made.

## Background

### Diabetes in pregnancy

The prevalence of diabetes in pregnancy is rising rapidly worldwide [1–5]. Diabetes pre-dating pregnancy (‘pre-gestational’) occurs in 0.5 % of pregnancies in Europe [6]. Gestational diabetes, defined as impaired glucose metabolism beginning in pregnancy, occurs in 2 % to 6 % of pregnancies [1] and is most common in women who are older, overweight, sedentary, have a family history of diabetes, had gestational diabetes in a previous pregnancy, or smoke [7–10]. However, the strongest risk factor for gestational diabetes is ethnicity, particularly South Asian, which confers a risk 6- to 11-fold higher than for white European or US women [11–13], though this drops to about 2.5-fold when other risk factors, particularly obesity, are included [14]. South Asian women account for only 9.2 % of all pregnancies in the UK and yet account for 25.6 % of pregnancies complicated by diabetes [15].

Women who have had gestational diabetes have a 7-fold risk of developing type 2 diabetes [16]. Children born to women with diabetes in pregnancy are more likely to develop type 2 diabetes in later life due to shared genetic and environmental risk as well as developmental programming [17], thus producing an intergenerational cycle of diabetes risk [18, 19]. Women can reduce their risk of diabetes by pre-pregnancy weight loss, adoption of a prudent diet, and regular physical activity [9, 20, 21]. The mainstay of management of diabetes in pregnancy (whether established diabetes or gestational) is tight blood glucose control, including pre-conception counselling where relevant. The package of care involves a multidisciplinary diabetes/antenatal team, self-blood glucose testing, education on “*the role of diet, body weight and exercise*” and appropriate medication when required (NICE guideline, February 2015) [22].

The immediate risks of diabetes in pregnancy to mother and child are potentially severe, including fetal loss, instrumental or caesarean delivery, prematurity, macrosomia, shoulder dystocia, and neonatal hypoglycaemia [13, 23–26]. These risks are mitigated significantly by good glycaemic control from 3 months prior to conception, throughout pregnancy and in labour [27, 28]. Achieving glycaemic targets requires strict adherence to lifestyle modifications (diet and exercise), medication, and attendance to antenatal services [29–34]. Such measures depend on maternal understanding and engagement, high-quality and timely education, and accessible and culturally acceptable services [32, 35, 36].

There is a large literature on cultural influences on the prevention and management of diabetes in South Asians [37–44]. Some South Asian women with diabetes in pregnancy engage reluctantly with health services and/or are diagnosed late [45–48]. Those with pre-gestational diabetes may have glucose control that is poor at conception and suboptimal throughout pregnancy [28, 49, 50]. The complex interaction of genetic, material, and socio-cultural influences can make diabetes in South Asians difficult to manage [3, 36, 51, 52]. South Asians in the UK are more likely to be affected by poverty, low health literacy, language barriers, and a tendency to prefer medication over lifestyle change [38, 50, 53–55]. These factors all shape the extent to which South Asian women engage in self-management and behaviour change strategies and attain the required glycaemic targets to reduce risk of maternal and fetal complications.

### A new theoretical framework

Notwithstanding the above literature, we believe that new theoretical models are needed to explore how the various demographic, behavioural, socio-cultural, and organisational influences on pregnancy outcome in South Asian women with dysglycaemia interact and build over time to influence successive pregnancies and future disease risk. We also need effective, theory-driven interventions that will improve the lifestyle choices women make before, during, and after pregnancy and the resources and support they can draw on when contemplating their options.

**Fig. 1:**
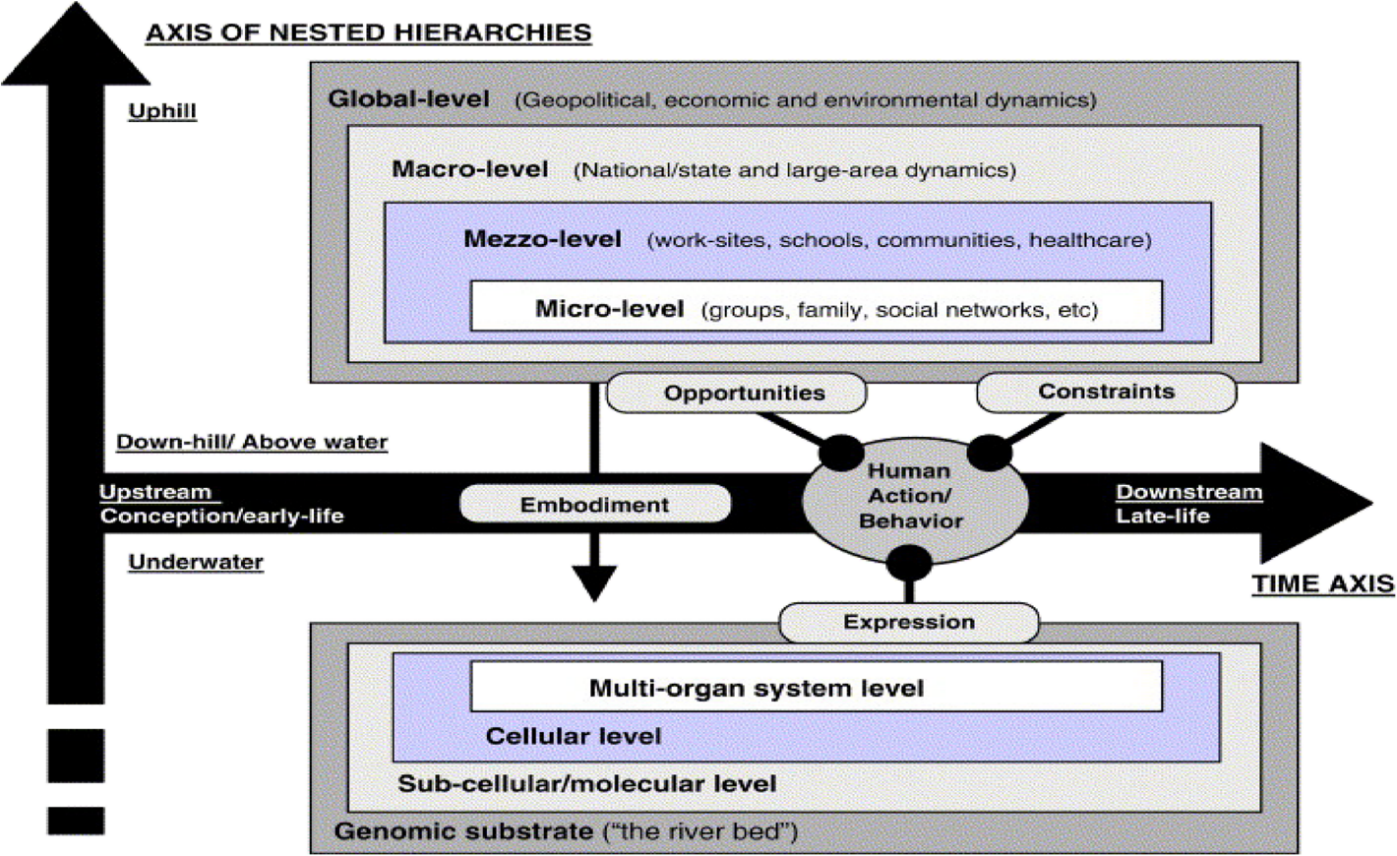
Glass and McAttee’s axis of nested hierarchies influencing behaviour and disease risk across the lifespan. Reproduced with permission from [58] *Caption from original*: The society-behavior-biology nexus as depicted in multidimensional space. The large arrows represent the axes of time and nested hierarchical structures. The sphere of health-related behavior and action moves through time from infancy to old age. Behavior is influenced by structures contingencies within the social and physical environment and by biological phenomena. Structural contingencies (opportunities and constraints) are shown by paths ending with nodes, while biological phenomena (embodiment and expression) are shown by paths ending with arrows or nodes

We began from the position that diabetes has anthropological and public health dimensions: its onset and course are strongly patterned by cultural practices and social determinants of health, including the material, cognitive, and socio-cultural effects of poverty, migration, education, and social capital [56]. Social ecology theory views health-related behaviours as the result of influences at multiple levels: intrapersonal, interpersonal, organizational, community, and public policy – hence, the most effective interventions tend to be multi-level [57]. Glass and McAttee, summarising previous work, proposed a ‘stream of causation’ flowing over time (the x-axis in Fig. 1) from upstream (in utero and early life exposures and windows of vulnerability) to downstream (later life manifestations) [58]. They further propose a nested hierarchy of systems from genes to cells and organs, the psychology of behaviour choices, the influence of social networks and groups, and the local and global environment (the y axis in Fig. 1), and a series of feedback loops and cross-level influences between these.

The question of how this nested hierarchy influences individual behaviour and therefore health outcomes is complex – and surprisingly under-researched. Amartya Sen’s health capabilities framework conceptualises health outcomes as the product of chances (the opportunities people have, which are economically, socially, and culturally determined) and the voluntary choices they make relative to these chances over their lifetime [59]. People from educated and affluent backgrounds have better chances but also typically have socialised dispositions that orient them to making health-positive choices [59]. Sociologists begin from the perspective that individuals’ actions and choices (which they tend to refer to as ‘agency’) both influence, and are influenced by, various influences in wider society (which sociologists refer to as ‘social structures’) such as prevailing rules, norms, cultural meanings, and material resources [60]. To date, theories of how societal forces link to individual behaviour have given limited attention to the genomic, biochemical, and physiological levels of influence (‘underwater’ in Fig. 1) – for example, how appetite from a low blood glucose level or a fright-flight response from an adrenaline surge might combine with external socio-cultural influences to produce particular behaviours in particular circumstances.

We sought to build on these multi-level, ecological models by using the narrative form to consider how people make sense of both external social structures and internal sensations and capabilities [61]. Long-term conditions such as diabetes produce both physical symptoms and impairments and biographical disruption: the sick and suffering person tells stories to build a coherent sense of self as they face an uncertain future [62]. In the illness narratives of migrants, personal experience is nested within a wider narrative of displacement, loss of social status, economic hardship, and perhaps discrimination [63]. Physical sensations and capabilities are culturally framed and sit within this nested hierarchy.

Narrative is a useful tool for capturing the richness of experience and placing it in historical, cultural, and personal context [64]. Potentially, the story form might surface hidden explanations about the reciprocal (i.e., mutually shaping) relationship between the different levels in the nested hierarchy in Fig. 1, including how human agency (behaviour, ‘choice’) is dynamically influenced by both the distal structural contingencies (external, ‘chances’) and the proximal biological embodiment (internal desires and drivers) described by Glass and McAttee [58].

## Methods

### Aim of the study

We hypothesised that the behaviour of women of South Asian origin before and during pregnancy would be shaped by physiological, practical, and cultural influences that would tend to compromise their own metabolic health and that of their offspring. In this in-depth qualitative study, we sought to i) understand in more detail the multiple influences on behaviour (and hence the risks to the metabolic health) of a South Asian mother and her unborn child; ii) theorise how these influences interact and build over time; and iii) inform the design of culturally congruent, multi-level interventions for individuals and communities.

### Management and governance

The study, which ran from 2012 to 2014, was part of an international collaborative programme of research, GIFTS (‘Genomic and lifestyle predictors of foetal outcome relevant to diabetes and obesity and their relevance to prevention strategies in South Asian peoples’, see [65]) with participating centres across Europe and South Asia. GIFTS was funded by European Framework 7 with additional support for this sub-study from the National Institute for Health Research. In addition to the over-arching GIFTS infrastructure, we set up a steering group for this London-based sub-project comprising research staff, clinicians, a research manager, and bilingual health advocates that met quarterly. Ethical approval was granted by Hampstead NHS Research Ethics Committee (12/LO/1418, November 2012 and subsequent amendments). Copies of invitation letters, information sheets, and consent forms are available from the authors.

### Study design

The study was performed through group story-sharing sessions and individual biographical life narrative interviews.

### Sample and setting

The study was based in two adjacent London boroughs, Tower Hamlets and Newham, both of which are ethnically diverse and have high levels of socio-economic deprivation. Participants were recruited from the Diabetes, Antenatal or Bilingual Health Advocacy and Interpreting Services at Barts Health acute trust. Inclusion criteria were Indian, Pakistani, or Bangladeshi ethnicity; diabetes, impaired fasting glucose, or history of gestational diabetes; fluency in one of the languages spoken by the research team (English, Urdu, Tamil, or Sylheti); and age over 18 and premenopausal.

### Story-sharing groups

The model of spontaneous, unstructured story-sharing in groups, facilitated by a bilingual health advocate [66], was inspired by our early qualitative study of illness narratives, which showed that Bangladeshis living in the UK linked positive lifestyle changes to stories told in informal settings by other Bangladeshis [39]. We have previously shown that, in adults from ethnic minority groups, group story-sharing was popular, increased people’s confidence and motivation for managing diabetes, and helped to make advice about lifestyle change meaningful [67, 68].

Women showing interest in this research were invited to join a story-sharing group, and those accepting were encouraged but not required to attend a course of six sessions. Participants in the Tamil and Gujarati (Urdu) groups usually preferred to speak English (though a bilingual researcher was present); those in the Bengali group spoke a combination of English and Sylheti, a dialect of Bengali. All group sessions were delivered by a bilingual health advocate trained in the sharing stories method [66]. Sessions lasted 2 hours; they were audiotaped with consent and transcribed. Sylheti sections were simultaneously translated and transcribed by a bilingual researcher (YC) and checked by a bilingual medical student (NA); discrepancies were resolved by discussion.

### Individual narrative interviews

Individual interviews were offered to women who had shown interest in the study but were unable to attend the group sessions (n = 28). For practical reasons (participant preference), all but four were held in the women’s homes. The biographical life narrative interview method, developed by Wengraf [69], was employed. The participant is asked to ‘begin at the beginning’ and tell their story uninterrupted. As in the group story-sharing sessions, the narrative approach was inherently phenomenological – that is, the focus of inquiry was perceptions and events that were of importance to the participants and experienced subjectively. We asked participants to talk generally about their life to date, including their family’s history of migration, and also more specifically about their diabetes and any past or hoped-for pregnancies. Prompts were kept to a minimum and were conversational in form (e.g., “tell me more about that”, or “what did you feel at that point?”).

### Data analysis

Data analysis (by TG, MC, YC, and NA) occurred in five overlapping stages: immersion, description, theorisation, illustration, and validation. In the immersion stage, all four researchers read and re-read transcripts to gain familiarity. In the description stage, NA, MC, and TG, working separately on Excel spreadsheets, used the framework approach to develop preliminary descriptive codes and categories [70]. Through discussion, this stage served to organise the data, increase our familiarity with it, and identify the different factors and influences relevant to living with diabetes, making positive lifestyle choices, and engaging with services, as previously described in detail [68]. Similarities and differences between ethnic groups were highlighted and discussed further.

**Fig. 2:**
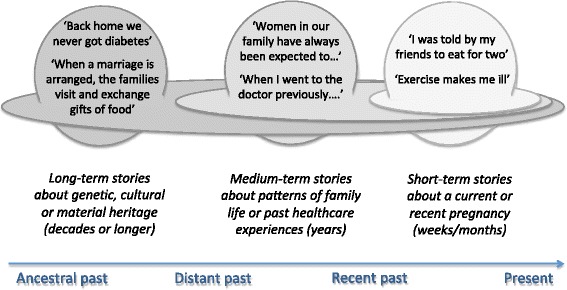
Schematic diagram of how narratives can capture interacting influences in the nested hierarchy model of health inequalities

In the theorisation stage, we drew on the theoretical perspectives described above, especially the nested hierarchy of systems shown in Fig. 1, to consider how and why individuals’ chances (socio-cultural and material), along with their embodied sensations and capabilities, influenced and interacted with their choices to produce health-positive or (more commonly) health-negative behaviours and outcomes. We worked partly from the Excel spreadsheets and partly by returning to group transcripts to consider stories in the context of group interaction. To capture the longitudinal and dynamic elements, we synthesised the data into a series of nested short-, medium-, and long-term ‘storylines’ – that is, abstracted accounts of how the lived experience of diabetic pregnancy unfolded over time in a woman of South Asian ethnicity living in the UK (Fig. 2). In each storyline, we began with individuals’ directly recounted stories of their short-term experiences, and then extended our analysis to consider how these experiences were shaped by longer-term and more distant influences that were evident in our data as story-fragments (for example as passing mention of relatives ‘back home’ or a comment on the price of food). As the analysis developed, we added successive findings to an emerging picture of the whole.

In the illustration stage, we used fictional-critical writing (that is, generating a new, fictional story based on findings from a number of real stories [71]) to generate vignettes that illustrated the salient influences and interactions between chances and choices. Finally, in the validation stage, the vignettes, along with the choices-chances interactions that underpinned them, were presented to the wider GIFTS steering group and refined where needed by discussion. The near-final vignettes were also presented to selected study participants to confirm that they rang true (what Bruner has called ‘verisimilitude’ [61]).

## Results

### Description of dataset

A total of 16 group story-sharing sessions were held, including 17 women in four groups, each of which met between three and six times. Participant characteristics are shown in Table 1. An early finding was that, although participants enjoyed the groups, they had difficulty attending. Recruitment was much slower than anticipated, mainly for practical reasons (women had childcare and other domestic commitments and nobody to ask for cover), and also because some women felt stigmatised by joining a group. Accordingly, a further 28 women were interviewed individually. The patient narrative dataset (audiotaped group and individual interviews) consisted of 82 pages of transcript. Staff interviews generated a further 75 pages of transcript.

**Table 1:** Participant characteristics

Language spoken at home	Bengali or Sylheti	Gurarati	Tamil	Punjabi or Urdu	Total
	(Bangladeshi origin)	(north-west Indian origin)	(south Indian or Sri Lankan origin)	(north Indian or Pakistani origin)	
Total number of participants	17	5	10	13	45
Median (range) age	37 (27–43)	34 (29–39)	34 (21–45)	35 (23–42)	35
Diabetes status					
Type 2	7	2	8	12	29
Gestational (past or present)	9	3	2	1	15
Missing data	1	0	0	0	1
Pregnancy status					
Median (range) number of pregnancies (including current one if relevant)	4 (2–9)	2 (1–4)	2 (2–4)	3 (1–6)	3 (1–9)
Pregnant at time of interview					
Yes	3	3	6	6	18
No	13	2	4	7	26
Missing data	1	0	0	0	1
Generation immigrant					
First generation	8	1	4	5	18
Second generation	7	2	3	6	18
Missing data	2	2	3	2	9
Group sessions					
Participated in group sessions	7	0	5	5	17
Median no. of sessions attended	3	n/a	2	3	3
Individual interviews					
Interviewed in clinic	0	2	1	1	4
Interviewed at home	10	3	4	7	24
Total	10	5	5	8	28

Gestational diabetes was diagnosed using locally-adapted UK criteria via universal 2 h, 75 g oral glucose tolerance testing of women of Asian origin at 28 weeks gestation with fasting plasma glucose ≥5.8 mmol/L and 120 min plasma glucose ≥7.8 mmol/L

### Experiences of pregnancy: common themes raised in the groups

The initial framework analysis identified a number of themes that were evident across all ethnic groups represented:

- Family life and domestic duties, which was depicted as busy, demanding, and tiring.
- Knowledge about pre-gestational and gestational diabetes. This was variable, with some participants well-informed but many ignorant of what diabetes is or how to prevent it, and confused about the distinction between type 1, type 2, and gestational.
- Beliefs and fears about diabetes in pregnancy – in particular, that having diabetes is dangerous for the unborn baby.
- Personal efforts to manage diabetes or the risk of gestational diabetes through lifestyle measures. These were usually depicted as unsuccessful and experienced as hard work, time-consuming, and stressful (“*when I try to diet I feel unwell and weak and have a headache*” *–* participant in Bangladeshi group 1)*.*
- Physical symptoms – especially, tiredness and lack of energy.
- Emotional symptoms – especially fear and a sense of lack of control (“*gotta go back and forth to the clinic, run tests, and you’re worried about your baby all the time*” – participant in Gujarati group).
- Perceptions and beliefs about local health services. These were largely positive and appreciative (*“There was more than enough help*” – participant in Bangladeshi group 2), though some commented that they had not attended antenatal or pre-conception services.

The framework analysis also identified some ‘silences’ in the data – themes that might have been expected but were not found. There were, for example, very few instances of personal strategies for continuing lifestyle change (such as plans to change diet, start exercising, or lose weight beyond the immediate period of pregnancy). In other words, what Whitmarsh calls the ‘ascetic subject of compliance’ (the individual who strives to comply strictly with medical advice on medication, self-monitoring, and recommended lifestyle choices) [72], was strikingly absent from our dataset. Furthermore, while many participants identified particular foods that they associated with being good or bad for health, very few participants grasped the significance of total energy intake in influencing body weight and/or metabolic risk.

In the next section, we build on these broad themes to illustrate through stories how multiple factors and influences, shown diagrammatically in Fig. 1, were depicted by our participants as interacting over time, at different levels, to create complex and often refractory preconditions for adverse health outcomes. The common storylines shared, which were evident across all ethnic groups studied, are shown in Box 1 and also illustrated in the fictional vignette in Box 2. As predicted by the ‘stream of causation’ and ‘nested hierarchy’ theories, these storylines had varying timescale and captured multiple interacting influences at different levels.

### Short-term storylines: the experience of pregnancy

Many participants depicted the experience of a current or recent pregnancy with pre-gestational or gestational diabetes as a stressful, ‘out-of-control’ state. While some women’s diabetes remained well controlled and asymptomatic throughout pregnancy, others never achieved stability in their blood glucose levels, hence experienced the entire pregnancy as physically tiring and anxiety-provoking. In order to be ‘in control’, these women said, they had to eat fixed amounts of particular types of food at fixed times, but achieving regular meals was difficult for the practical reasons discussed below. These narratives seem to reflect the significant challenge in achieving tight glycaemic control and the multiple factors, including the composition of food intake and the complexity of glucose and insulin dynamics, experienced by these women with diabetes in pregnancy [73].

“*I don’t know how, like, my* [non-diabetic] *sister can feel so good when pregnant. You can’t do this, can’t do that, I didn’t enjoy my pregnancy.*” *–* Participant in Urdu Group, Session 2.

Some women emphasised the destabilising influence of nausea and/or vomiting in early pregnancy. The task of self-administering insulin injections was experienced by some as painful and emotionally traumatic.

Another common short-term storyline was “*exercise makes me ill when I’m pregnant*”. Many, though not all, participants avoided exercise during pregnancy because, they felt, exercise seemed to destabilise their condition further, causing muscle pain, breathlessness, swollen feet, sweating (experienced as unnatural and unpleasant), faintness, dizziness, or profound tiredness. Some described a vicious circle of exercise avoidance leading to physical deconditioning, weight gain, and a worsening of diabetic control, as illustrated in Box 2. These stories resonated with previous research on South Asian women [45], but contrasted strikingly with those collected in other studies on pregnant middle class white women, who depicted yoga, swimming, or gym as contributing effectively to the control of back pain and improving wellbeing and energy levels (e.g., the HealthTalk resource of pregnancy stories [74]).

Many women in our groups considered housework, taking children to and from school, and – for Muslim participants – prayer (that is, five times daily physical prostration) to be a form of exercise. They felt that having done these duties, there was neither time nor need for formal exercise regimens. The few who described planned exercise saw this as a short-term fix for raised blood glucose levels “*I would eat around seven and then walk for a bit, I would climb up and down the stairs a few times and that’s when I would feel it came to a normal level*” *–* Bangladeshi Interviewee 0033*.*

The storyline of ‘eating for two’ in pregnancy was common. Eating was depicted as giving strength to the mother and providing the unborn baby with essential nutrients, without which it would not grow properly. Conversely, stories of restricting food intake (sometimes in response to advice from a health professional) were almost invariably linked to negative physical effects in the mother (headaches, fainting, lack of energy) and perceptions of poor growth in the fetus. Food was depicted as providing positive nourishment rather like a short-term dose of good medicine, and not as potentially harmful or metabolically destabilising “*Even in food there is medication, you have to eat a small portion of rice*” *–* Bangladeshi Interviewee 0034.

Participants did not generally view weight gain in pregnancy as an indication that they should cut back on their food intake for three reasons: belief in food as essential nourishment; attribution of the weight gain to changes other than obesity (‘bloating up’); or linking weight gain (perhaps rightly) to commencing insulin. They generally sought to avoid unpleasant bodily symptoms and nurture their unborn baby, accepting deterioration in their diabetic control as a necessary trade-off. Some viewed cutting back on food as dangerous*.*

Another common short-term storyline was advice from relatives or peers. Older family members were often knowledgeable in how to manage diabetes and encouraged self-discipline in diet or exercise. Female relatives advised on how to manage the pregnancy in ways that did not always resonate with medical advice. Some participants placed higher value on advice from peers than on professional advice. As one put it, “*A lot of people advised me to eat this or eat that for your diabetes so I followed their orders rather than just the doctors*” – Participant in Bangladeshi Group 1, Session 2.

A striking feature of these short-term storylines was how rarely women appeared to resist social pressures, tolerate uncomfortable physical symptoms, or challenge advice offered by others. This might be explained in terms of cognitive and emotional dispositions – that is, the psychological states and traits that impair an individual’s ability to cope. These traits differed across our sample but commonly included an external locus of control (belief that diabetes is caused by things outside her own control), low levels of self-efficacy (belief in one’s own ability to complete tasks and reach goals), and low health literacy (limited knowledge and understanding of the condition and the healthcare system). Such dispositions were commonly though not universally evident in our sample, as illustrated in Box 2, and they meant that even when a woman’s chances were not entirely adverse, her choices were unlikely to maximise health outcomes.

### Medium-term storylines: family life and past experience

The lives of most participants were characterised by domestic and maternal duties that were not merely demanding but (perceived as) never-ending. They typically spent many hours every day doing housework, entertaining in the home, and taking and collecting children (their own or other people’s) from school. As one domestic task ended, another began. In almost all families, domestic duties appeared to be the exclusive province of women, and the young mother held a particularly pivotal and subservient role in the kinship group. During one interview at a participant’s home, guests arrived unannounced. The participant immediately diverted her attention to cooking a generous meal for the guests, and resumed the interview after they had eaten and left.

Importantly, and in contrast to the stereotype of the extended Asian family, many women in our sample had limited family support, hence domestic duties over-rode even serious health problems. One described an antenatal consultation at which she was told she must be immediately admitted to hospital with dangerously high blood pressure but being unable to comply because she had no-one to pick up her son from school.

Mealtimes were determined by the patterns of work and other commitments of family members, with the implication that the young wife and mother would adjust her own routine to fit these “*I wait until my husband gets back, between 8 and 9 pm, and have dinner with him*.” – Participant in Urdu Group, Session 2.

The cultural, practical, and material constraints of the traditional South Asian wife and mother role severely limited participants’ opportunities for attending to personal health and in particular for taking physical exercise. Long periods of time were spent in the kitchen, where sweets kept for entertaining guests and leftovers from family meals were a constant temptation “*all I’ve become is a waste bin*” – Participant in Bangladeshi Group 1, Session 3.

Our visits to women’s homes to conduct individual interviews revealed multiple material and social stressors that formed a context for these medium-term storylines: several participants (who had been unable to attend group sessions) lived in cramped, squalid conditions in high-crime areas, and some were in short-term tenancies with the threat of eviction. These women’s accounts of ‘exercise’ (shopping, taking children to school) were heavily overlaid with concerns about safety for themselves and their children.

Medium-term storylines also illustrated the significant role of past experience in shaping individual dispositions, both physical and cognitive-emotional. Some had been overweight for several years, struggling with progressive weight gain and/or worsening glucose tolerance during and after previous pregnancies. Such stories were often nested in narratives about neighbourhood and community conditions including the walkability of the built environment and availability and cost of food. Many foods recommended by health professionals (low-energy, low glycaemic index, high nutritional value) were depicted as expensive, while fast food (high-energy, low nutritional value) was seen as ubiquitous and affordable.

In terms of the experiences that shaped cognitive-emotional dispositions, many participants had had little relevant health education or experience, partly because of the young age at which motherhood began (see example in Box 2). Most participants who had taken insulin had done so only during pregnancy, hence had had little or no previous experience of managing injections or flexible dosing. These findings contrasted with our previous research on older South Asians who had (or were at risk of) diabetes, many of whom had good basic knowledge and understanding of the condition and (where relevant) were proficient and confident in administering insulin [37, 68].

A key aspect of past experience that shaped women’s response to their current pregnancy was past illnesses and/or encounters with the health service. In general, they spoke positively of hospital services, especially incidents in which they were given medication for an acute condition that was cured or alleviated as a result. Some had had negative experiences of general practitioners, who (allegedly) had not listened to them, not treated them with respect, enforced a ‘one appointment, one problem’ rule, or told them to change their lifestyle rather than prescribe a medicine. These negative experiences were sometimes cited as the reason why a woman delayed presenting to her GP when she fell pregnant.

### Longer-term storylines: genetic and cultural heritage

As the vignette in Box 2 illustrates, the short-term storyline of pregnancy as an ‘out of control state’ is nested not only in the medium-term storyline of domestic roles, socialised dispositions, and obesogenic local environment described in the previous section but also in the longer-term meta-narratives of genetic, cultural, religious, and economic heritage.

One such meta-narrative is the Western notion of selfhood, within which arguments about personal ‘behavioural choices’ – and Whitmarsh’s ‘ascetic subject of compliance’ [72] – make (more or less) sense. As Mol has argued, non-Western cultures have a more collective ethos within which such choices make far less sense (and in which it is much less possible to be ‘compliant’) [75]. The description by South Asian women of their work as (non-negotiable) ‘never-ending’ contrasts, for example, with what has been called the Taylorisation of domestic time (that is, counting the hours spent on domestic duties and seeking to make this expenditure more efficient and gender-equal) among middle-class Western women [76].

The widely-held assumption that housework was adequate exercise, which women readily agreed went against health professional advice, is likely to have arisen historically at a time when women’s domestic role also included fetching water and (for example) harvesting rice from local fields (though the better-off would have had servants for these tasks). The expectation that leftovers will be consumed by the women of the house ‘behind the scenes’ outside normal mealtimes is partly linked to a longer-term storyline of food insecurity and under-nutrition of women, in particular at a time when food was less plentiful and men’s nutritional needs were prioritised [77]. It has become acutely counterproductive in modern times when ‘leftovers’ may consist of high-energy entertaining food including sweets, biryani rice, and fatty meat.

The storyline of frequent and highly influential (but not always correct) advice from female peers and relatives, tellingly referred to as ‘orders’ in the quote above, is nested in a longer-term storyline of pregnancy and childbirth as a traditional rite of passage, managed by and among women within a particular South Asian culture [43–46, 78]. The common storyline of expecting medicine, and of treating particular foods as ‘medicine’, rather than taking responsibility for more enduring lifestyle change, can be understood partly in terms of historical patterns of predominantly acute, infectious illness (such as malaria or polio) in South Asian countries [79]. The concept of chronic, life-long incurable disease (or risk factors for disease) caused by an excess of food and requiring restraint from the patient is a more recent (hence less tenacious) storyline – despite the fact that diabetes was accepted as very common in the British South Asian community.

## Discussion

### Summary of findings

This study has sought to explain the poor health outcomes for both mother and offspring linked to pre-gestational and gestational diabetes in South Asians. We have documented the various influences on behaviour during and between their pregnancies, at an individual (experiences, dispositions, actions), family (roles, routines, expectations, disposable income), community (environment, local services), and societal level (e.g., national and international economic context, health policy, and the collective history of migration and memories of life ‘back home’).

We have used narrative to track the ‘streams of causation’ posited by Glass and McAttee’s nested hierarchy approach [58], and hence have begun to capture how such South Asian women’s practices are affected over time by these multiple interacting influences. These can lead in the short term to complications in pregnancy, in the medium term to increased risk of type 2 diabetes in the mother, and potentially, in the longer term, to adverse metabolic programming in the offspring. It is hoped that advances in molecular science will allow integration of this nested hierarchy with novel insights into the genomic, metabolic, and physiological processes involved in diabetes, pregnancy, and their complications.

**Fig. 3:**
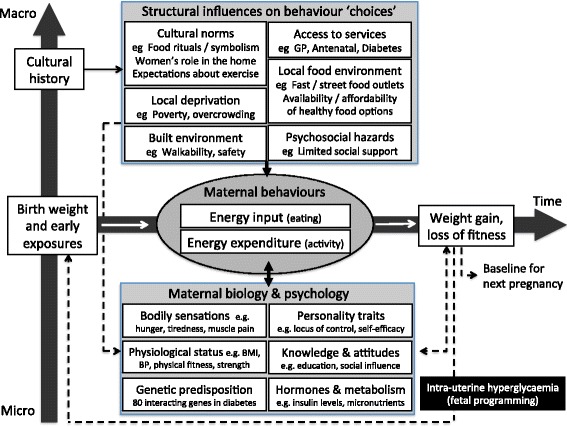
Schematic diagram of the intergenerational transmission of diabetes risk via nested hierarchy of influences around pregnancy. Adapted from Glass and McAttee [58]

Figure 3 shows a schematic diagram of how the multiple nested influences coalesce to influence two key health ‘choices’ before, during, and after pregnancy: food intake and physical activity. This diagram was adapted from Glass and McAttee’s illustration of how mediating structures in the family and community affect the expression of risk factors and disease across the life course. As these authors emphasise, such structures (which they also refer to as ‘risk regulators’) should not be thought of deterministically as causes of poor health outcomes, but they help explain the accumulation and distribution of causes. They are “*the bridging tendrils linking larger macro-social processes (such as systems of stratification, labor markets, culture, and systems of production and migration) to the behavioral sphere of human activity and decision making*” ([58], p. 1660).

### Comparison with other studies

Our findings resonate strongly with those of previous researchers, who have shown that some South Asian women hold negative views about exercise (especially during pregnancy), consider that their domestic role is sufficient physical exertion, perceive the environment for exercising (streets, parks, leisure facilities) to be unsafe or culturally unwelcoming, and report practical or comorbidity barriers to taking exercise [37, 38, 45]. Previous studies have also demonstrated the cultural significance of traditional foods and ritual meals in South Asians and the central role of the wife and mother in cooking and entertaining guests [37, 40, 41]. A study of South Asian women in Canada showed that, whilst women knew about the antenatal education classes, not many attended because they were receiving advice from family or friends, they had no time, their husband was not around, there was a language barrier, or they did not have any transport [47, 48].

In a previous qualitative study on the food beliefs of Bangladeshis in London, we found two interrelated and intersecting binary systems of food classification, ‘strong’/‘weak’ and ‘digestible’/‘indigestible’, based upon the belief that all foods have two aspects of being: a physical substance or ‘body’ (*gator* or *sharir*) and a life energy or ‘strength’ (*shakti*) [79]. A food’s nourishing power was seen as lying hidden in the food’s physical substance and separable from it through digestion; the latter was then excreted as waste. Participants in that study frequently likened the body to a mechanical machine, and talked of food as ‘the body’s fuel’. The Bangladeshi folk model of food was thus of something that had more or less positive (strength-giving) substance and greater or lesser digestibility but no negative substance. This may explain the lack of attention given by our participants to the potential adverse effects of excess energy intake.

What the current study adds to all the above literature is a richer theorization of how the multiple influences, proximal and distal, interact via the final common pathway of human behaviour to produce a progressive accumulation of adverse metabolic consequences, which operate over the life course. The hierarchy of cultural and practical influences is a complex web from which few traditional South Asian women can break out, as illustrated by the vignette in Box 2. We have also gone beyond previous studies to show how bodily sensations, especially hunger and non-specific feelings of tiredness and body pains (which are real but also culturally framed and inextricably entangled with roles and expectations within the family [56, 80]), can drive a vicious circle of negative health choices, especially excess energy intake and low physical activity levels in pregnancy, storing up metabolic trouble for both mother and fetus.

### Limitations of this study

The main limitation of this study is the relatively small sample size and the poor attendance at the story-sharing groups (though on another level, this finding was important data that underlined the heavy and non-negotiable domestic commitments of this target group). Whilst 45 is a large sample for a qualitative study, and we did not detect striking differences between the different South Asian ethnic groups, numbers were insufficient to support definitive conclusions about inter-group commonalities and differences. We invite others to replicate the study in different contexts.

### Implications for policy and practice

The findings of this study affirm previous work that the intergenerational cycle of morbidity resulting from diabetic pregnancy is mediated by the behaviour and lifestyle choices of the mother (especially how much she eats and how much energy she expends). Our findings also demonstrate that those choices are strongly influenced by bodily symptoms and drivers (including a woman’s baseline level of fitness and the labile nature of glucose metabolism in some South Asian pregnancies), and are nested within family, community, and wider social structures (deeply held cultural norms, beliefs and rituals, and the material constraints of poverty, housing, and the built environment) that are not easily changed.

It follows from our findings and the wider literature reviewed above that educational interventions that focus exclusively on individual education (and which assume that behaviour change will follow logically from the acquisition of knowledge) are doomed to fail – not least because they presuppose an ‘ascetic subject’ for whom compliance with behaviour change would be a realistic possibility [72]. Nevertheless, some areas of significant misunderstanding need addressing – notably, the finding that many women in our sample appeared to view diabetes as either ‘present’, ‘absent’, or ‘gone away’ and saw food as a short-term source of strength and exercise as a short-term fix for raised blood glucose. They had little understanding of the progressive and enduring metabolic damage associated with excess energy balance in the presence of non-modifiable risk factors for diabetes.

For health education to be successful, however, we need to make explanations and interventions more meaningful. The behaviours of the South Asian mothers in our sample made sense to them and to their families, and were viewed (rightly or wrongly) as health-giving. Using Whitmarsh’s terminology, pregnancy-related behaviours and the peer advice that often prompted them were intimate (that is, deeply personal), familiar (that is, grounded in the richness of family relationships and traditions), and seen by the women and their families as the right thing to do [72]. In contrast, conventional health education addresses intimate themes but takes little account of why particular behaviours are meaningful or morally resonant; instead, it seeks to engineer choices that are abstract, unfamiliar, lacking in clear moral justification, and which might even seem perverse.

The argument from some women in our sample “*I was advised* [by peers] *that I should eat… so I ate*” resonates with our previous finding in Bangladeshis that stories recounted by peers had a powerful influence on health-related behaviour [39]. The power of peer advice to influence behaviour even when it cuts across professional recommendations may be partly due to the fact that such advice (especially when presented through a storytelling format) is familiar, meaningful, and morally grounded rather than unfamiliar, abstract, and morally rootless. To be effective, educational interventions need to emulate these principles – that is, advice should reflect and be attuned to the short, medium, and long-term drivers that generate particular behaviours by particular individuals in particular cultural contexts; behavioural options need to be presented as meaningful possibilities and take account of what is (and is not) seen as right and reasonable behaviour.

The group sessions offered in this study were poorly attended, though those who did attend evaluated them highly. In randomised trials, group education programmes for self-management of diabetes in South Asians, when well attended, culturally tailored, intensively delivered in a community setting, and linked to clinical care planning can produce significant improvements in control [81, 82]. Trials of group-based antenatal education in non-diabetic women in low-resource settings have shown significant improvements in maternal and fetal mortality in low-resource settings [83]. Trial populations, however, may be atypical [84]. Group-based peer support through storytelling is one way in which health education can be made familiar and meaningful by making it both personal and culturally grounded, and by providing a forum for negotiating the micro-morality of lifestyle choices (that is, what it is best to do in a particular set of circumstances) [68]. However, if women of childbearing age find it impossible, practically or culturally, to attend face-to-face groups, these theoretical benefits will be hard to realise in practice.

Many young women of South Asian origin are regular users of computers, so there is some potential for using the virtual environment to provide group education and peer support. However, virtual story-sharing is a departure from historical and cultural traditions, and we do not know if such a medium would be either acceptable or meaningful to women. More promising, perhaps, is a community-based service model in which diabetes antenatal education and support is provided alongside childcare and opportunities for social interaction. Such a model would resonate with the wider chronic care model, which proposes that the prevention and management of chronic illness requires interaction between i) informed, active patients or citizens; ii) engaged, proactive healthcare teams mindful of cultural traditions; and iii) prepared and adequately-resourced communities [85, 86].

## Conclusions

As Glass and McAttee comment, human behaviour is “*sandwiched inextricably between ecology and biology*” ([58], p. 1656)*.* The high and rising prevalence of diabetic pregnancy and its adverse short- and longer-term consequences in South Asians, particularly those who migrate to high-income countries and regions, are mediated by patterns of human behaviour (what women eat, and how much exercise they take, before and during pregnancy) that are poorly matched to their metabolic needs [87]. This so-called match-mismatch paradigm has long been recognised as the root of the escalating epidemic of diabetes and its intergenerational transmission. In sum, diabetes develops because of inadequate physiological and metabolic responses to “*chronic fuel excess, which results in so-called nutrient spillover, insulin resistance, and metabolic stress*” ([52], p. 169).

The molecular, cellular, and pathophysiological pathways through which energy imbalance does damage to mother and offspring are now well established, and understanding of the genetic basis of this process is expanding rapidly. Genetic and molecular explanations are important, but as Famer et al. commented in their classic paper on structural violence, “*exclusive focus on molecular-level phenomena has contributed to the increasing ‘desocialization’ of scientific inquiry: a tendency to ask only biological questions about what are in fact biosocial phenomena*” ([88], p. 1686).

Epigenetics offers promise to redress this bias, but the socio-cultural mechanisms that generate adverse patterns of human behaviour, and the reasons why these patterns are so resistant to change, have, to date, only been factored into the epigenetic model at the most speculative level. For example, Nolan et al. state: “*Improvements in maternal public health programmes in pre-transition and post-transition populations and provision of education to relevant groups about the risks of rapidly adopting western lifestyles could be considered*” ([52], p. 176).

This study has extended Nolan et al.’s important work to produce a much richer theorisation of the socio-cultural levels of influence and how these interact with the more micro levels in Fig. 1 [52]. Addition of sociological and anthropological theories to the model has revealed why ‘education about risks’ is unlikely to be effective, and has highlighted the need for models of education and clinical support that are defensible anthropologically and pedagogically as well as physiologically. Further research should develop and test such models empirically.

## Box 1: Common ‘storylines’ shared in groups or raised by individuals

Short-term (stories about a current or recent pregnancy)

- Pregnancy with diabetes as a stressful, out-of-control state
- Impact of behaviour on symptoms, especially ‘exercise makes me ill’ and ‘I feel better when I eat for two’
- Accounts of advice, especially from other women

Medium-term (stories about family life, community life, past healthcare encounters)

- Stories of domestic life, especially ‘a woman’s work is never done’
- Stories of progressive weight gain
- Past experiences with illness and/or health services, e.g., ‘when I went to the doctor I was told my illness was not important’

Long-term (stories about the distant past)

- Genetic heritage, e.g., strong family history
- Cultural heritage, e.g., subservience of the individual to family/community
- Material heritage, e.g., food insecurity, 74/75 famine in Bangladesh

## Box 2: Fictional-critical vignette of a South Asian woman with diabetes in pregnancy

Fatima is 31. She came to the UK from Bangladesh at 16 and married at 17. She had five children – three girls and then two boys. Her last child, born 3 years ago, was stillborn at 36 weeks. All her children weighed more than 3.5 kg. Her elder son, delivered by forceps, spent three days in the special care unit when he was born. Fatima’s eldest daughter Ratna is now 13 and is teased at school for being overweight.

Fatima says she did not have diabetes with her first three pregnancies (though she’s not sure if she was tested). She was diagnosed with gestational diabetes at 24 weeks in the last two pregnancies. She was terrified when told this diagnosis, fearing that the unborn baby would be damaged or die. She was told to cut down her rice intake, avoid sugar and sweet foods, and use less oil. She tried hard to follow this advice, but the diet made her feel very weak and her female friends and relatives told her she must take sufficient rice to maintain her strength and nourish the baby.

In her last two pregnancies, Fatima monitored her sugar levels by pricking her finger several times a day. She attended the hospital clinic every week, taking her younger children with her on the bus. The staff were very nice but the clinic was busy and waiting was stressful. Later in each pregnancy, Fatima was put on insulin injections, which she hated as they were painful. She persevered because she feared for her child’s life. Her father, who lived nearby, was able to help and support her with the insulin injections because he too was taking insulin. But Fatima felt that her dad’s diabetes was easy to control compared to her own, which seemed to fluctuate unpredictably.

Fatima felt very tired, and her feet swelled up, especially during her last two pregnancies. Her regular domestic duties (housework, cooking, taking the children to and from school) took many hours and were physically demanding, as was her five times daily prayer ritual. She was sure all this exercising made the tiredness much worse, so she tried to rest up as much as possible between her chores. In any case, she did not feel safe exercising outdoors in the evenings in this part of town.

In each of her pregnancies, Fatima put on weight, which she thought was due mainly to fluid. She lost some but not all of this weight after the baby was born – perhaps partly because she found herself finishing up leftovers (her family cannot afford to throw food away). Her mother and sister had had a similar pattern of progressive weight gain with each of their pregnancies, so she felt this was probably normal.

Thankfully, a blood test 6 weeks after the birth of each of Fatima’s sons showed that her diabetes had gone away. She was glad she did not have it any more and that she could eat normally with the family again. She was very surprised a year later when her tiredness returned and her GP diagnosed type 2 diabetes. She cannot understand this, since she never took sugar in her tea after attending the diabetes education sessions when she was pregnant, and she uses less oil in cooking than most of her friends. The GP told her she must go back on the strict diet, take tablets, and take more exercise – otherwise she might get complications. But with four children and elderly in-laws to look after at home, she has very little time to attend to her own health.
